# Interactions between MFAP5 + fibroblasts and tumor-infiltrating myeloid cells shape the malignant microenvironment of colorectal cancer

**DOI:** 10.1186/s12967-023-04281-6

**Published:** 2023-06-21

**Authors:** Zhiwei Peng, Zihao Ren, Zhiwei Tong, Yinan Zhu, Yansong Zhu, Kongwang Hu

**Affiliations:** 1 Department of General Surgery, First Affiliated Hospital of Anhui Medical University, Hefei, 230022 Anhui China; 2 School of Life Science, Anhui Medical University, Hefei, 230022 Anhui China; 3 Department of General Surgery, Fuyang Affiliated Hospital of Anhui Medical University, Fuyang, 236000 Anhui China

**Keywords:** Single cell RNA-sequencing, Spatial transcriptomics, Colorectal cancer, Macrophages, Fibroblasts, MFAP5, C1QC

## Abstract

**Background:**

The therapeutic targeting of the tumor microenvironment (TME) in colorectal cancer (CRC) has not yet been fully developed and utilized because of the complexity of the cell–cell interactions within the TME. The further exploration of these interactions among tumor-specific clusters would provide more detailed information about these communication networks with potential curative value.

**Methods:**

Single-cell RNA sequencing, spatial transcriptomics, and bulk RNA sequencing datasets were integrated in this study to explore the biological properties of MFAP5 + fibroblasts and their interactions with tumor-infiltrating myeloid cells in colorectal cancer. Immunohistochemistry and multiplex immunohistochemistry were performed to confirm the results of these analyses.

**Results:**

We profiled heterogeneous single-cell landscapes across 27,414 cells obtained from tumors and adjacent tissues. We mainly focused on the pro-tumorigenic functions of the identified MFAP5 + fibroblasts. We demonstrated that tumor-resident MFAP5 + fibroblasts and myeloid cells (particularly C1QC + macrophages) were positively correlated in both spatial transcriptomics and bulk RNA-seq public cohorts. These cells and their interactions might shape the malignant behavior of CRC. Intercellular interaction analysis suggested that MFAP5 + fibroblasts could reciprocally communicate with C1QC + macrophages and other myeloid cells to remodel unfavorable conditions via MIF/CD74, IL34/CSF1R, and other tumor-promoting signaling pathways.

**Conclusion:**

Our study has elucidated the underlying pro-tumor mechanisms of tumor-resident MFAP5 + fibroblasts and provided valuable targets for the disruption of their properties.

**Supplementary Information:**

The online version contains supplementary material available at 10.1186/s12967-023-04281-6.

## Introduction

Colorectal cancer (CRC) is one of the most common malignancies, with more than 1.9 million new cases and over 935,000 deaths reported globally in 2020 [1]. Multiple studies have investigated CRC pathogenesis. Many therapeutic strategies targeting cancer cells have also been developed. However, tumor cell-based treatment is still associated with multiple problems including tumor metastasis, recurrence, and therapy resistance, which require further research [2]. The conceptual shift from a tumor cell-centered model to a tumor microenvironment (TME)-centric model broadens the field of tumor biology. It also provides possible solutions for these problems [3, 4].

The tumor microenvironment is comprised of complicated non-cancerous cellular components, including immune cells, fibroblasts, and endothelial cells, as well as acellular products, such as soluble factors and extracellular matrix (ECM) [2]. Cancer-associated fibroblasts (CAFs) and tumor-infiltrating immune cells are the most common stromal cells observed within the TME. Recent studies have increasingly demonstrated the critical role of the extensive crosstalk between CAFs and immune cells in tumor development [5]. However, the previously deciphered tumor promotion or suppression mechanisms of CAF-immune cell interactions have not yet been fully elucidated and utilized in focused therapy because of limited technology in the past. With the rapid development of single-cell RNA sequencing technology (scRNA-seq), we can now determine the details of heterogeneous TME biological properties at single-cell resolution [6, 7]. As scRNA-seq cannot be used to explore spatial tissue architecture, we are still unable to precisely decipher complex cellular interactions that occur throughout the tissue space. Spatial transcriptomics technology (ST), which has emerged recently, enables the exploration of spatial gene expression and cellular arrangements within multicellular organisms. Therefore, the combination of single-cell technologies with spatial transcriptomics could enable the detection of the heterogeneous cell population details as well as providing insight into spatial tissue organization [8, 9].

Interactions between CAFs and immune cells have been widely investigated previously. However, the identity of these cells still remains elusive because of the complexity of the TME. Therefore, further study is required. Hence, in the present study, we utilized a multi-omics strategy, including scRNA-seq and ST, to comprehensively analyze the crosstalk between tumor-infiltrating fibroblasts and myeloid cells involved in CRC. We particularly focused on the functions of the previously rarely investigated MFAP5 + fibroblasts, which were identified in CRC tissues. We observed that the TME alters the biological behavior of tumor-resident MFAP5 + fibroblasts, conferring them with a pro-tumor tendency. Through in-depth analyses, we also identified for the first time that the close localization of MFAP5 + fibroblasts and tumor-associated macrophages, especially C1QC + macrophages (previously identified macrophages with pro-inflammatory role), [10] could facilitate the tumor invasive phenotype. We were also able to confirm that the mutual interactions between MFAP5 + fibroblasts and tumor-infiltrating myeloid cells prompt malignancy by activating pro-tumorigenic signaling pathways, such as the MIF/CD74 and IL34/CSF1R axes in myeloid cells, as well as enhancing the aggressive phenotypes of MFAP5 + fibroblasts through EGF and VISFATIN signals in a positive loop. Therefore, these cell–cell interactions could be targeted to develop an optimal strategy for CRC therapy.

## Methods

### Data sources

In this study, we integrated six independent public datasets that contained single-cell RNA sequencing data (downloaded from Gene Expression Omnibus (GEO) repository: https://www.ncbi.nlm.nih.gov/geo/query/acc.cgi?acc=GSE144735) [11], spatial transcriptomics data (10X Genomics) (derived from a previous published research at website: http://www.cancerdiversity.asia/scCRLM/) [12], and four bulk transcriptomics of colon and rectal cancer (downloaded from TCGA cohort COAD and READ at website: https://xena.ucsc.edu/; GSE14333 and GSE17536 cohorts were downloaded from GEO repository).

### Single-cell RNA sequencing analysis

The R (v4.0.5) package Seurat (v4.0.2) [13] was used to process the scRNA data. As dataset quality control had been performed in previous studies, we did not further filter the scRNA-seq data. The SCTransform method was used to normalize the data. The harmony method (v0.1.0) was applied to remove batch effects and integrate the Seurat objects into a single dataset [14]. The integrated data were subjected to a principal component analysis (PCA) algorithm to reduce the data dimensions. FindNeighbors and FindClusters were used to cluster cells with similar characteristics. A uniform Manifold Approximation and Projection (UMAP) algorithm was used for data visualization.

### Differential expression analysis, cell annotations and enrichment analysis

Differentially expressed genes (DEGs) of cell populations in scRNA-seq were identified using FindAllMarkers. Genes that were positively expressed in more than 25% of the cells in each cluster were selected. Of which, canonical cell markers, top 20 DEGs of each cluster, and CopyKAT algorithm (infer tumor or normal epithelial cells) [15] were combined for cell annotations. In order to analyze the functions of each cluster, the R package clusterProfiler (v3.18.1) was used for GO enrichment analysis [16]. We set* p* < 0.05, |avg_logFC|> 1 as the cutoff criteria.

### Spatial transcriptomics data analysis

The R package Seurat was also used for spatial transcriptomics data (ST) processing and visualization. In order to normalize the ST data, we used the SCT method; the functions SelectIntegrationFeatures, PrepSCTIntegration, FindIntegrationAnchors, and IntegrateData were used to integrate the ST data. An unsupervised clustering method was subsequently used to cluster similar ST spots. Cell population annotations were based on hematoxylin and eosin staining (HE) sections and the highly variable genes in each cluster. Scores of cell-specific signatures (top 20 DEGs) from scRNA-seq were calculated using two methods: ssGSEA algorithm and AddModuleScore function. SpatialDimPlot and SpatialFeaturePlot were combined to visualize the cell expression level in the ST data.

### Cell-type infiltration analysis

In order to calculate the cell-type infiltration levels in ST data, TCGA cohort COAD, and READ, we integrated the top 20 DEGs identified in scRNA-seq as well as previously well-defined immune cell gene sets [17]. We also calculated the scores of M1/M2 signatures using the ssGSEA algorithm [18] with the GSVA package (v1.38.2) [19]. To visualize the spatial organization of MFAP5 + fibroblasts and C1QC + macrophages in ST slices, we scored the top 20 DEGs of cell clusters using the AddModuleScore function. To comprehensively analyze the differential expression of MFAP5 between tumors and adjacent normal tissues in diverse cancer types, we used the online tool TIMER (https://cistrome.shinyapps.io/timer/ [20].

### Correlation analysis

Correlation analysis was performed to investigate the interactions between specific cells. The R packages ggstatsplot (v0.10.0) and corrplot (v0.92) were used for data analysis and visualization [21]. In addition, the online tool TIMER was used to establish the correlation between gene expression and immune infiltration levels in the colon and rectal cancer cohorts [20]. *p* < 0.05 was considered as statistically significant.

### Survival analysis

In order to explore the role of specific cells in clinical diagnosis and prognosis, we used Survival (v3.2-10) and Survminer (v0.4.9) to conduct survival analysis on the COAD, READ, GSE14333, and GSE17536 cohorts. The cell population infiltration (the top 20 DEGs identified in scRNA-seq) was calculated using the ssGSEA algorithm. The median was selected as the cutoff value to differentiate patients into distinct groups (high or low). The Kaplan–Meier survival curve was modelled using the function survfit. Subsequently, a Cox proportional hazards regression model was established to determine the independent risk factors. In addition, PrognoScan was used to explore the prognostic value of MFAP5 using three independent databases [22].* p* < 0.05 was considered as statistically significant.

### Cell-to-cell communications analysis using CellChat

The CellChat (v1.1.3) method was used to infer cell-to-cell interactions between fibroblasts and myeloid cells and to build a regulatory network based on ligand-receptor crosstalk [23]. We filtered out cell–cell communications that were expressed in fewer than 10 cells in certain cell groups. The netVisual function was used to visualize interaction patterns. When signaling pathways with more than one ligand-receptor pair were evaluated, we used the netAnalysis_contribution function to compute and visualize the contribution of each ligand-receptor pair to the overall signaling pathway. PlotGeneExpression was used to visualize the expression of ligands and receptors of certain signaling pathways in cell populations using violin plots. Non-negative matrix factorization (NMF) algorithms were considered when selecting the number of co-communication patterns in the CellChat object. This was after utilizing the identifyCommunicationPatterns function, which was adopted to identify major signals for certain cell populations and general communication patterns. In addition, netAnalysis_teCentrality was used to calculate network centrality scores, and netAnalysis_signalingRole_network was used for visualization. These were used to identify dominant senders, receivers, mediators, and influencers in certain inferred networks.

### Immunohistochemical staining analysis

Immunohistochemistry (IHC) was performed to detect cell-type-specific protein expression in CRC. Tissue microarray (TMA) (Cat. No. IWLT-N-70C42) consisted of 35 pairs of tumor and para-tumor samples that were obtained from Wuhan Servicebio Technology Co., Ltd. TMA was first treated with a boiling antigenic repair solution (EDTA, pH9) for 15 min. Subsequently, 3% hydrogen peroxide was applied to inactivate endogenous peroxidases. Following that, the slices were blocked in 3% BSA for 30 min. After adding primary antibodies (MFAP5, Rabbit, Cat. No. DF13146, Affinity, 1:50), they were stored for incubation overnight at 4 ℃. The following day, the sections were washed three times with PBS and incubated with an HRP-conjugated secondary antibody (Cat. No. GB23303; Servicebio, 1:200) for 50 min at room temperature. Diaminobenzidine (DAB) was used as the chromogen and nuclei were stained with hematoxylin. Double immunohistochemical staining for MFAP5 (Rabbit, Cat. No. DF13146, Affinity, 1:100)/CD163 (Mouse, Cat. No. EM1901-90, HuaBio, 1:100) and IL-34 (Rabbit, Cat. No. DF13820, Affinity, 1:100)/CSF1R (Mouse, Cat. No. EM1708-56, HuaBio, 1:100) was performed while following the instructions of the immunohistochemical double staining kit (ZSGB-Bio, Cat. No. DS-0004). HRP/AP conjugated secondary antibodies were mixed for incubation for 30 min at room temperature, and DAB and AP-red were used as chromogens. In order to objectively quantify the protein expression of MFAP5 in tumors and adjacent normal tissues, the H-score was used as a semiquantitative indicator [24]. H-score (histochemistry score) is a histological scoring method for processing immunohistochemistry. The number of positive cells and their staining intensity in each section are converted into corresponding numerical values to achieve the purpose of semi-quantitative tissue staining. H-Score = ∑(pi × i) = (percentage of weak intensity × 1) + (percentage of moderate intensity × 2) + (percentage of strong intensity × 3), where pi represents the proportion of positive signal pixel area/cell number; i stands for coloring intensity. For data with H-score between 0 and 300, larger data indicate stronger comprehensive positive rate [25]. To compare the H-score of paired cancer tissues and para-cancer tissues, we applied paired-sample Wilcoxon test for statistical analysis, when we compared the H-score among different TNM stages, we used Wilcoxon test to compare the differential expression of MFAP5 in different TNM stages.

### Multiplex immunohistochemistry assay

Multiplex immunohistochemistry (mIHC) method was used to detect three different antibodies in the sections. Primary antibodies included MFAP5 rabbit anti-human antibody (cat. No. DF13146, Affinity, 1:3000), C1QC rabbit anti-human antibody (Cat. No. BS-11337R; Bioss, 1:200), and EPCAM mouse anti-human antibodies (cat. No. GB12274, Servicebio, 1:200). Cancer tissues or adjacent normal tissues used for the mIHC experiment were obtained from previously collected paraffin-embedded surgical specimens of patients with colorectal cancer, who had signed informed consent forms. A TSAPLus Fluorescence Triple Staining Kit (Cat. No. G1236-50T, Servicebio) was used to stain the primary antibodies. All the experimental procedures were performed according to the manufacturer’s instructions (Service Bio). On the first day, we repaired the antigen, inactivated endogenous peroxidases, blocked the antigen, and labeled the sections with the first primary antibody MFAP5 at 4 ℃ overnight. On the second day, after labeling with 488-TSA fluorescence (1:500), the tissue sections were treated with an antigenic repair solution. Subsequently, we repeated the previous steps and incubated the sections with two primary antibodies, C1QC and EPCAM at 4 ℃ overnight. On the third day, the sections were stained with CY3-(1:200) and CY5-(1:200) and conjugated with secondary antibodies for 50 min. The nuclei were stained with DAPI.

## Results

### Single-cell transcriptomics atlas of heterogeneous tumor microenvironment in CRC

**Fig. 1:**
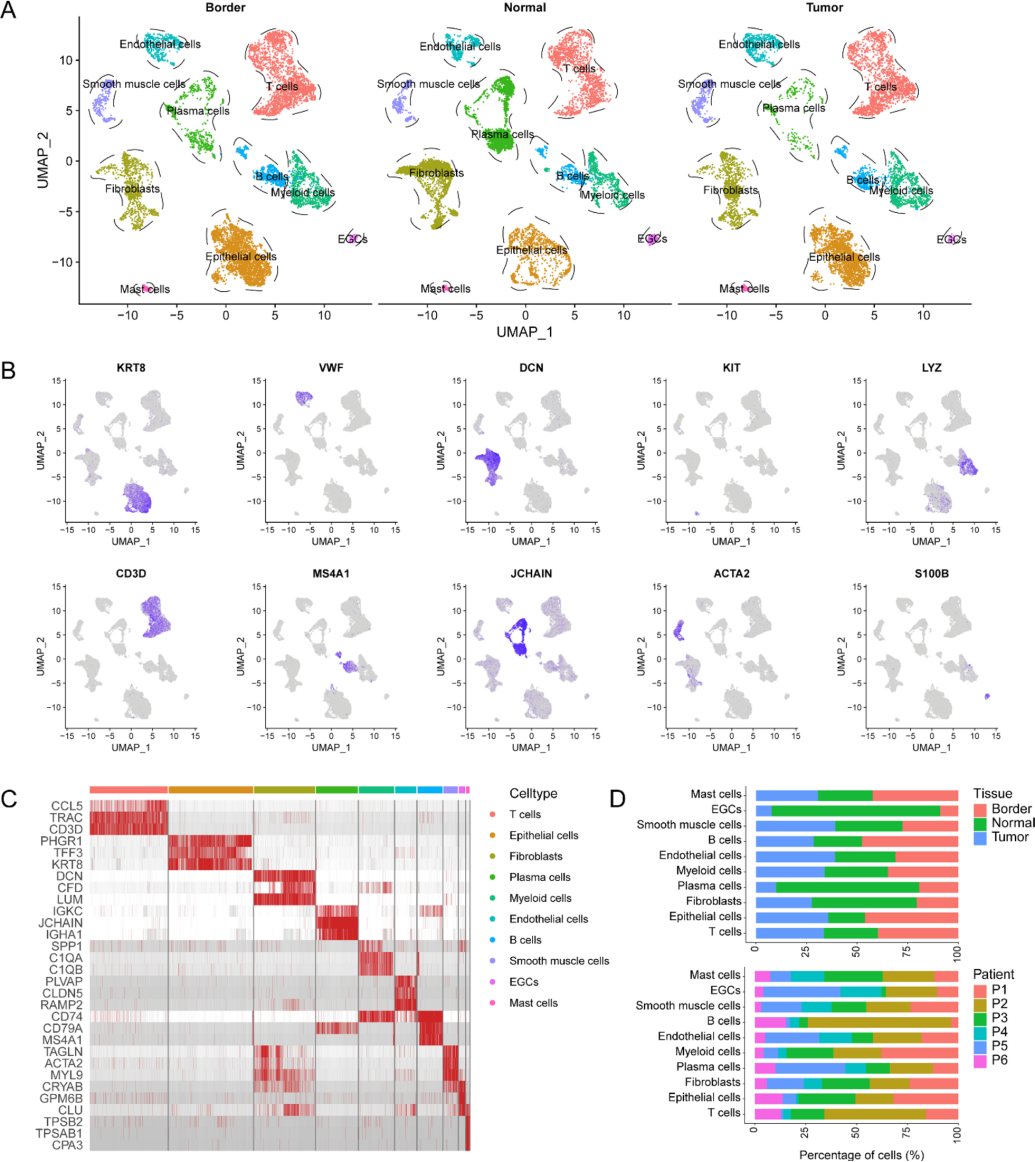
Single-cell transcriptomics atlas of heterogeneous tumor microenvironment in CRC. **A** UMAP plots of scRNA-seq profiled in this project faceted by tissue types (include tumor core, border and adjacent normal tissues). **B** Feature plots of canonical cell-type signatures expression in each cluster. **C** Heatmap visualizes the top 3 differentially expressed genes (DEGs) of each cellular population. **D** Cell infiltration level in diverse tissues (upper) or patients (lower) inferred by scRNA-seq

In order to define the heterogeneous single-cell landscapes of CRC, we used a previously published single-cell dataset containing six paired samples from tumor core regions, border tumor regions, and matched normal mucosa [11]. A total of 27,414 cells were included in subsequent analyses. These included 8254 cells from tumor core regions, 9424 cells from tumor border regions and 9736 cells from adjacent normal tissues. The cell populations were defined into ten cell types based on previous well-defined gene markers [12, 26–31]: epithelial cells (n = 6243) were identified by high expression of KRT8 and EPCAM; endothelial cells (n = 1573) express VWF and ENG; fibroblasts (n = 4498) exhibit high expression of DCN, COL1A1 and COL1A2; mast cells (n = 255) were marked by KIT; myeloid cells (n = 2613) were identified by LYZ expression [29]; T cells (n = 5757) were marked by CD3D and CD3E expression; cell populations with high expression of MS4A1 were identified as B cells (n = 1847); plasma cells (n = 3089) were identified by JCHAIN [31]; smooth muscle cells (n = 1070) were marked by ACTA2; and a small number of cells with a high expression of S100B were considered as enteric glial cells (EGCs, n = 469) (Fig. 1A–C and Additional file 1: Table S1) [24]. The infiltration of the ten cell types was different in distinct tissues. We applied CopyKAT algorithm to infer tumor cells from normal epithelial cells. As we had expected, the majority of tumor-derived epithelial cells are malignant tumor cells, while in matched normal mucosa only contains normal epithelial cells (Additional file 1: Fig. S1). B cells, myeloid cells, and T cells had higher infiltration levels in tumor regions (including core and border) than in normal tissues. However, plasma cells demonstrated lower infiltration in these regions. This could reflect the heterogeneous microenvironment and different stages of tumor progression (Fig. 1D).

### Tumor-specific microenvironment shapes the malignant properties of tumor-associated MFAP5 + fibroblasts

**Fig. 2:**
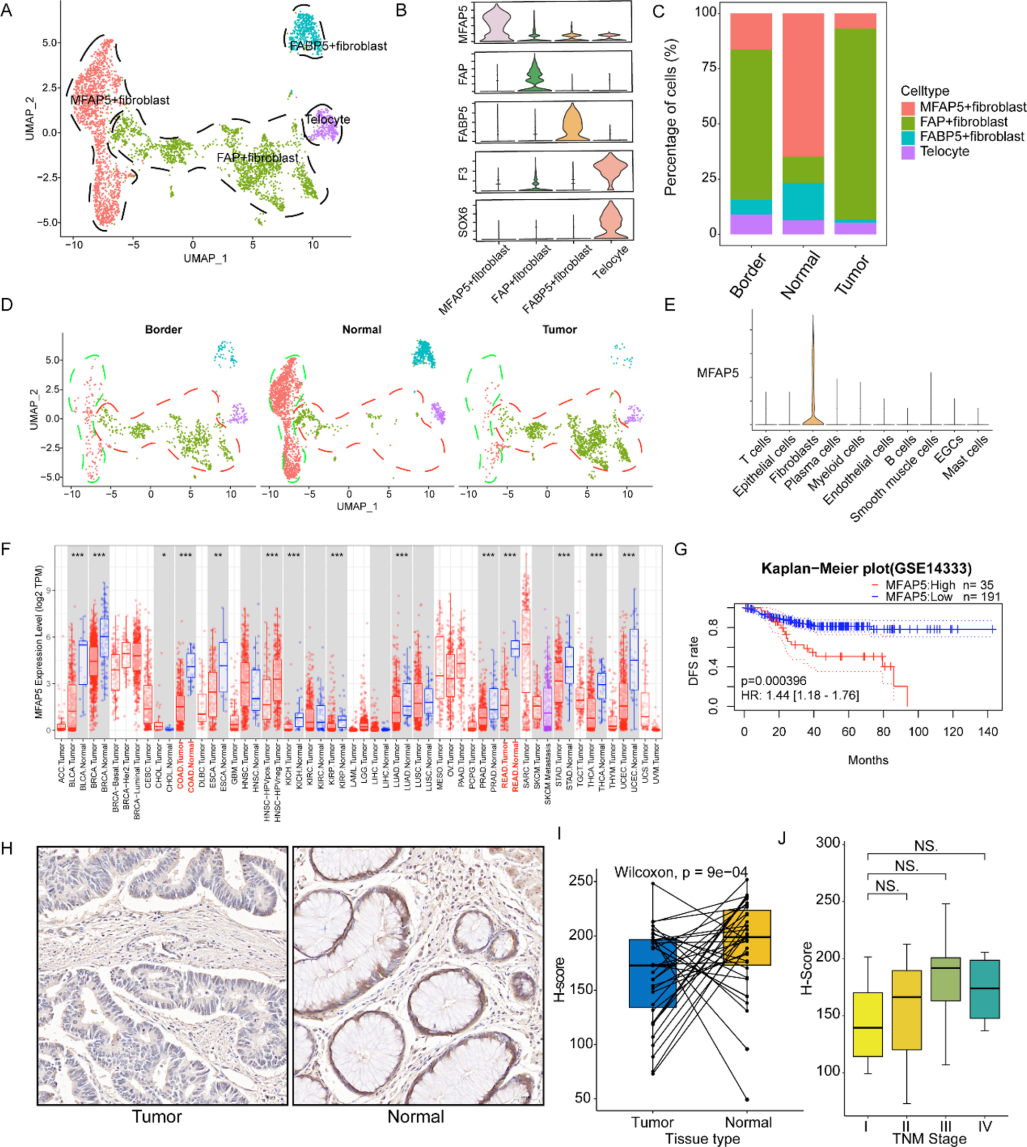
Tumor-specific microenvironment shaped the malignant properties of tumor-associated MFAP5 + fibroblasts. **A** UMAP plot of scRNA-seq of fibroblasts in CRC. **B** Violin plot shows the cell markers of specific fibroblast subclusters. **C** Stacked bar plot of inferred cell proportions of fibroblast subpopulations in different tissues. **D** UMAP plots of fibroblast subclusters faceted by tissue types (include tumor core, border and adjacent normal tissues). **E** Violin plot suggests that the MFAP5 is a fibroblast-specific signature in CRC. **F** Pan-cancer analysis of MFAP5 expression between cancer and para-cancerous tissues visualized by TIMER tool. **G** Kaplan–Meier survival plot indicates that CRC patients with high level of MFAP5 have worse clinical outcome in GSE14333 cohort. **H** IHC analysis show the existence of MFAP5 + fibroblasts in tumor and normal tissues. **I** Quantification of MFAP5 staining intensity in the tumor and normal sites with H-score, paired-sample Wilcoxon test used for statistical analysis. **J** Box plot shows the correlation between H-score and TNM stage, Wilcoxon test applied to compare the differential expression of MFAP5 in different TNM stages, *p* < 0.05 as statistically significant

Fibroblasts are the most abundant stromal cells. They are also considered the key cell type involved in regulating tumor progression [24]. Multiple studies have investigated the biological behavior of cancer-associated fibroblasts. However, because of the heterogeneity of the microenvironment, the identity of these cells remains elusive [24, 32, 33]. In order to further clarify the heterogeneity of fibroblasts, we combined the reported canonical fibroblast markers, functional enrichment, and highly variable features of each cluster for cell annotation (Fig. 2A, B, Additional file 1: Figs. S2, S3, and Additional file 1: Tables S2, S3). MFAP5 + fibroblasts were characterized by high MFAP5 expression. Fibroblasts marked by a canonical fibroblast activation signature (FAP) were identified as FAP + fibroblasts [24]. Telocytes are known to express SOX6 and F3 [34]. We also identified a fibroblast subtype (FABP5 + fibroblasts) with high expression of FABP5 and FABP4, which were co-expressed in microvascular endothelial cells [35]. Therefore, we speculated that FABP5 + fibroblasts were derived from endothelial cells which might have undergone an endothelial-to-mesenchymal transition and acquired fibroblast-like phenotypes.

Next, we compared the grade of infiltration for each fibroblast subtype in distinct tissues and observed that FAP + fibroblasts mainly existed in tumor regions, including the cancer core and border. In contrast, MFAP5 + and FABP5 + fibroblasts were notably present in normal tissues. This could be attributed to their different biological characteristics (Fig. 2C, D) [36]. Moreover, it is worth noting that MFAP5 is a cell type-specific signature that is principally expressed in specific fibroblast subtypes residing in normal tissues (Fig. 2C–E). Subsequently, we applied the online tool TIMER [20] to investigate the pan-cancer differential expression of MFAP5 between tumor and normal tissues. The results demonstrated that MFAP5 was upregulated in almost all normal tissues of diverse tumor types, particularly in COAD and READ (Fig. 2F). We subsequently examined the association between MFAP5 + fibroblasts and clinical prognosis. We observed that the higher the expression of MFAP5, the poorer the survival outcome of patients with CRC across the three independent public cohorts (Fig. 2G and Additional file 1: Fig. S4). We then applied tissue microarray (TMA) of 35 pairs of CRC tissues to confirm this (Fig. 2H). Our findings again suggested the higher expression of MFAP5 in normal tissues as compared with that in tumor sites (Fig. 2I). The IHC analysis suggested that the higher the H-score, the higher the TNM stage (Fig. 2J), which implies stronger capability for invasion and metastasis. However, there was no statistical difference, which could be attributed to the small sample size in TMA. We speculated that TME-specific factors, such as bioactive molecules, complicated intercellular interactions, and stimuli, contribute to the alteration of the biological properties of tumor-resident MFAP5 + fibroblasts confers these cells with a tumor-promoting tendency. However, this is not observed in normal-resident fibroblasts.

### Multi-omics analyses uncover the cell-to-cell communications between MFAP5 + fibroblasts and macrophages

**Fig. 3:**
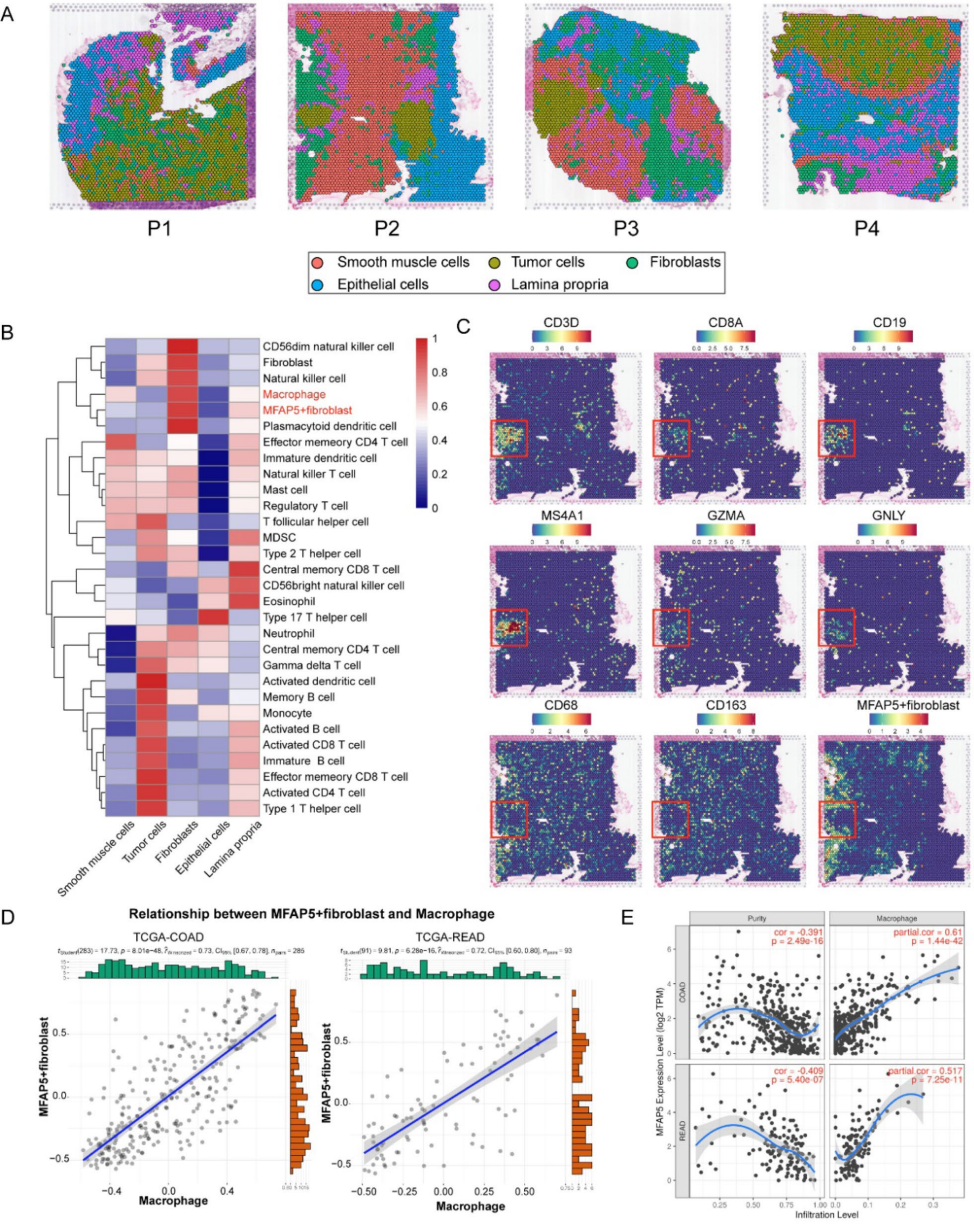
Multi-omics analyses uncover the cell-to-cell communications between MFAP5 + fibroblasts and macrophages. **A** Spatial images show the tissue architecture of CRC inferred by unsupervised clustering method. **B** Heatmap of immune cells and fibroblasts infiltration level scored by ssGSEA algorithm. **C** Gene expression of CD3D, CD8A, CD19, MS4A1, GZMA, GNLY, CD68, CD163 as well as MFAP5 + fibroblasts (top 20 DEGs identified in scRNA-seq) in spatial organization of patient 2 (P2), red boxes mark the representative tumor region of CRC. **D** Scatter plots show the positive correlation between MFAP5 + fibroblasts and macrophages in COAD and READ cohorts. **E** Correlation analysis of MFAP5 and tumor-infiltrating immune cells shows the highest correlation between MFAP5 and macrophages calculated by TIMER tool

The reciprocal interactions between fibroblasts and immune cells during cancer progression have been widely investigated during recent years [24, 37, 38]. Hence, we hypothesized that abnormal crosstalk between MFAP5 + fibroblasts and other tumor-infiltrating immune cells could be an important cause of the aberrant biological behavior demonstrated by MFAP5 + fibroblasts in the CRC microenvironment. In the present study, we used spatial transcriptomic data to assess the spatial organization of MFAP5 + fibroblasts and immune cells in CRC. Based on the HE stained sections and DEGs of each cluster, we annotated the spatial spots into five main clusters: tumor cells, fibroblasts, smooth muscle cells, epithelial cells, and lamina propria (Fig. 3A). First, we used the ssGSEA algorithm to score the signatures of MFAP5 + fibroblasts (top20 DEGs) and immune cells (well-defined gene markers, see details in Additional file 1: Table S4) [17] in ST (Fig. 3B). We detected the co-enrichment of MFAP5 + fibroblasts and macrophages in the fibroblast region, whereas some adaptive immune cells such as B cells and T cells were scarce in the same regions (Additional file 1: Table S5). We used another method, AddModuleScore, to score the enrichment of MFAP5 + fibroblasts. We observed that MFAP5 + fibroblasts mainly infiltrated fibroblast areas that were close to M2-phenotype macrophages (marked by CD68 and CD163) but opposite to anti-tumor T cells or B cells (Fig. 3C and Additional file 1: Fig. S5). We also scored the MFAP5 + fibroblasts and macrophages within the public TCGA cohorts COAD and READ. Based on the correlation analysis, a significant positive correlation could be verified between them (Fig. 3D). Finally, we examined the correlation between MFAP5 gene expression and immune cell infiltration and observed that among all immune cells, MFAP5 demonstrated the highest correlation with macrophages (Fig. 3E and Additional file 1: Fig. S6). These results highlight the existence of extensive cell-to-cell interactions between MFAP5 + fibroblasts and macrophages, which may play a crucial role in regulating CRC progression within the TME.

### Cell–cell interactions of MFAP5 + fibroblasts and C1QC + macrophages are associated with poor CRC prognosis

**Fig. 4:**
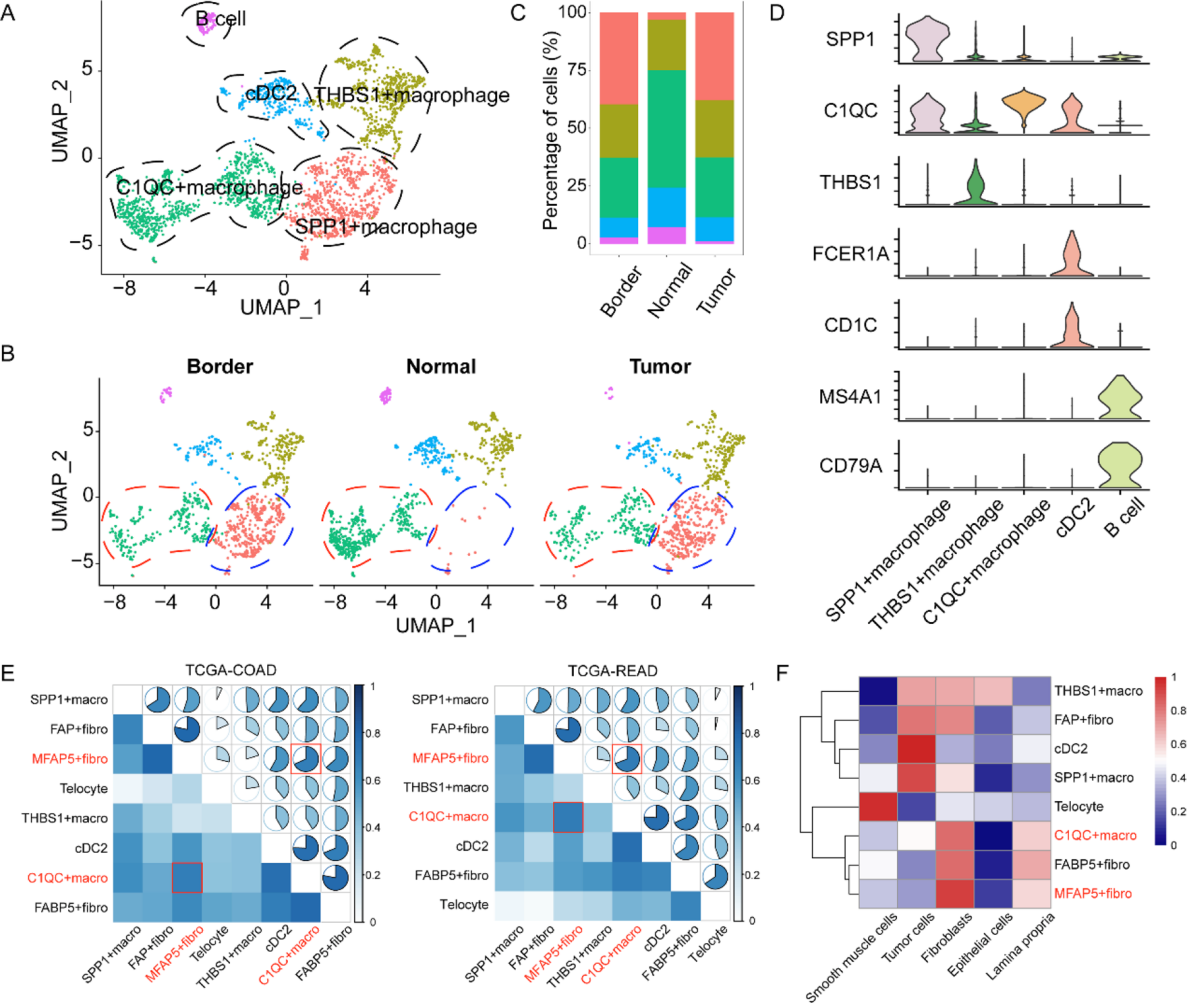

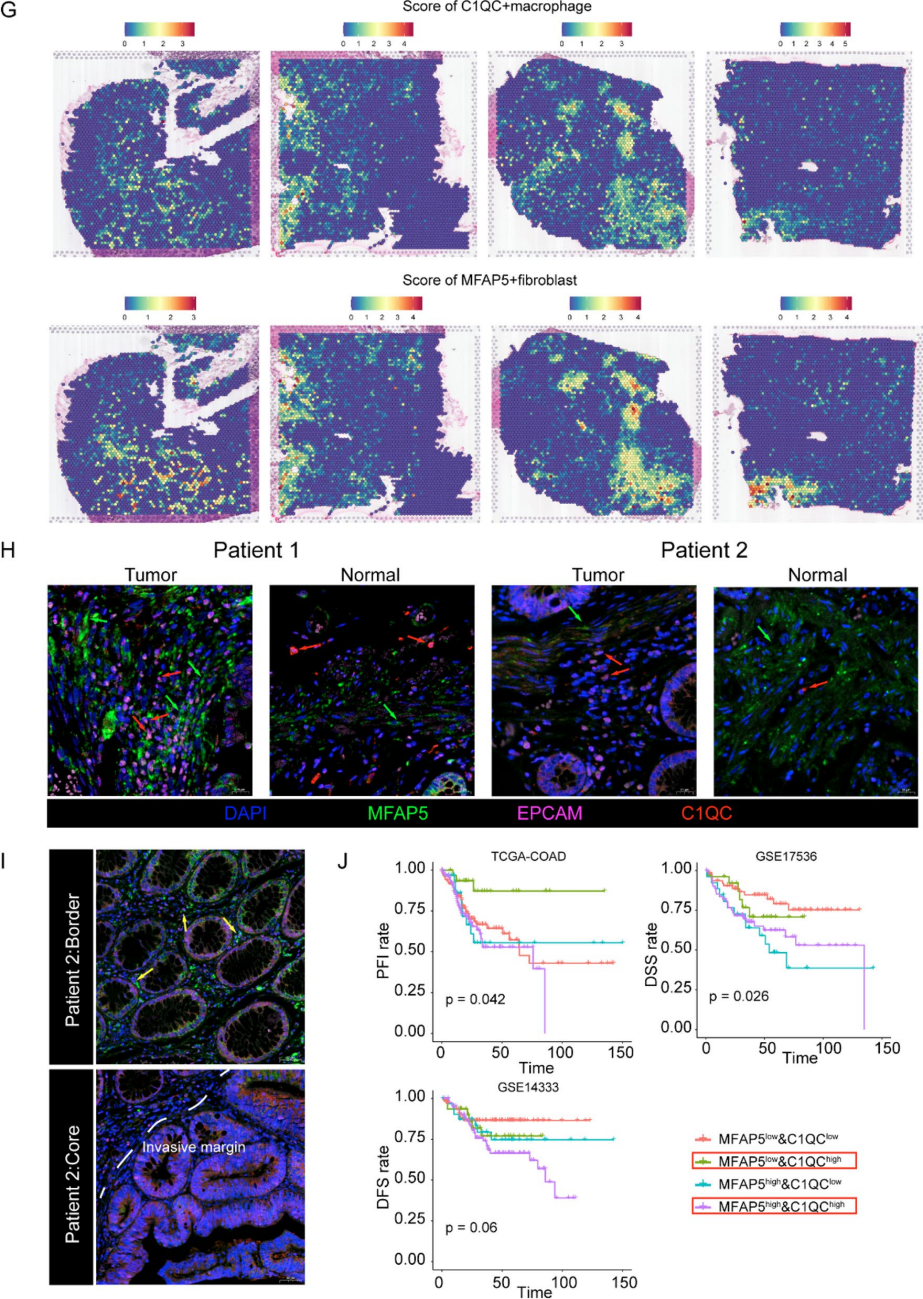
Cell–cell interactions of MFAP5 + fibroblasts and C1QC + macrophages are associated with poor CRC prognosis. **A** UMAP plot of tumor-infiltrating myeloid cells colored by cell types. **B** UMAP plots of myeloid cells subclusters faceted by tissue types (include tumor core, border and adjacent normal tissues). **C** Stacked bar plot of myeloid cell proportions in different tissue types. **D** Violin plot of cell-specific signatures of myeloid cells. **E** Complex heatmaps exhibit the correlation between tumor-associated fibroblasts and myeloid cells in two independent cohort: COAD and READ, the correlation between MFAP5 + fibroblasts and C1QC + macrophages marked with red box. **F** Heatmap of fibroblasts and myeloid cells infiltration level scored by ssGSEA algorithm in ST. **G** Spatial transcriptomics images of MFAP5 + fibroblasts and C1QC + macrophages scored by AddModuleScore. **H** mIHC figures show the localization of MFAP5 + fibroblasts and C1QC + macrophages in tumor or normal tissues of Patient 1 and Patient 2, red arrows show the representative C1QC + macrophages, green arrows represent MFAP5 + fibroblasts (×40, 20 μm). **I** mIHC figures show the co-localization of MFAP5 and C1QC + macrophages in the tumor core or border regions in Patient 2, yellow arrows show the microfibrils adhered C1QC + macrophages (×20, 50 μm). **J** K–M plot shows survival analysis for both expression of MFAP5 + fibroblasts and C1QC + macrophages in TCGA-COAD, GSE14333 and GSE17536 cohorts

Myeloid cells are the leading tumor-infiltrating immune cells with tumor-suppressive or tumor-promoting functions [10]. Reclustering of myeloid cells was classified into four myeloid cell subtypes and B cells (MS4A1^high^ and CD79A^high^) (Fig. 4A–D, Additional file 1: Fig. S7, and Additional file 1: Table S6). Dendritic cells (DCs) with high expression of FCER1A and CD1C are recognized as cDC2 [10]. Macrophages with high expression of THBS1 are characterized as THBS1+ [24]. In addition, classical SPP1 + macrophages were also identified with positive expression of SPP1 in our study [10, 24, 39]. We observed that tumor-associated macrophages highly expressing C1QC, C1QA, and C1QB (Fig. 4D and Additional file 1: Fig. S6) could be identified as C1QC + macrophages. This finding was similar to that observed in a previous study [10]. As analyzed previously, we demonstrated the close proximity of MFAP5 + fibroblasts and macrophages in CRC. However, the specific types of macrophages that interact with MFAP5 + fibroblasts remain unclear as yet. Therefore, we used TCGA datasets to determine the correlation between MFAP5 + fibroblasts and myeloid cells identified in our study. The results demonstrated a significant positive correlation between the infiltration of MFAP5 + fibroblasts and C1QC + macrophages (Fig. 4E, Additional file 1: Tables S7, S8). The ST data were subsequently analyzed to validate the relationship between these cells. Two algorithms, ssGSEA and AddModuleScore, were used to calculate the signature scores of these cells. The results indicated that MFAP5 + fibroblasts and C1QC + macrophages were co-enriched in the fibroblast region (Fig. 4F, G and Additional file 1: Table S9). Furthermore, the mIHC assay confirmed the co-localization of MFAP5 + fibroblasts and C1QC + macrophages within the tumor regions. In contrast, there were few C1QC + macrophages around the MFAP5 + fibroblasts in normal tissues, suggesting that crosstalk between these cells mainly occurred in tumor tissues (Fig. 4H and Additional file 1: Fig. S8). MFAP5 is a protein coding gene which could remodel the ECM by the synthesis of elastic microfibrils [40]. We observed that MFAP5 + fibroblasts-derived microfibrils could adhere to the C1QC + macrophages in tumor border stromal region rather in tumor invasive margin (Fig. 4I and Additional file 1: Fig. S9). This was also reflected in ST slices (Fig. 4G and Additional file 1: Fig. S10). We hypothesized that this phenomenon could facilitate its signal communication with C1QC + macrophages in border local stroma to a certain extent [5].

A previous study has reported that high infiltration of C1QC + macrophages results in high immune cell infiltration [41] and good prognosis, which could be attributed to the pro-inflammatory functions of C1QC + macrophages [10]. We assessed the clinical value of C1QC + macrophages in four independent CRC cohorts. We observed that patients with low infiltration of MFAP5 + fibroblasts and high levels of C1QC + macrophages experienced better prognoses. In contrast, patients with high expression of both cell types demonstrated poor outcomes in the three cohorts (Fig. 4I). After adjusting for sex, age, and pathologic stage using the Cox proportional hazard regression model, we observed that compared with the MFAP5 + fibroblasts^high^ and C1QC + macrophages^high^ groups, patients with lower MFAP5 + fibroblast infiltration and higher C1QC + macrophage infiltration demonstrated better prognoses (Additional file 1: Fig. S11). Hence, we hypothesized that aberrant signal communication between MFAP5 + fibroblasts and C1QC + macrophages promotes the tumor-invasive phenotype to a certain extent. We carried out subsequent analyses to confirm this finding.

### Fibroblasts-myeloid cells based regulatory network detect tumor-specific signaling pathways in CRC

**Fig. 5:**
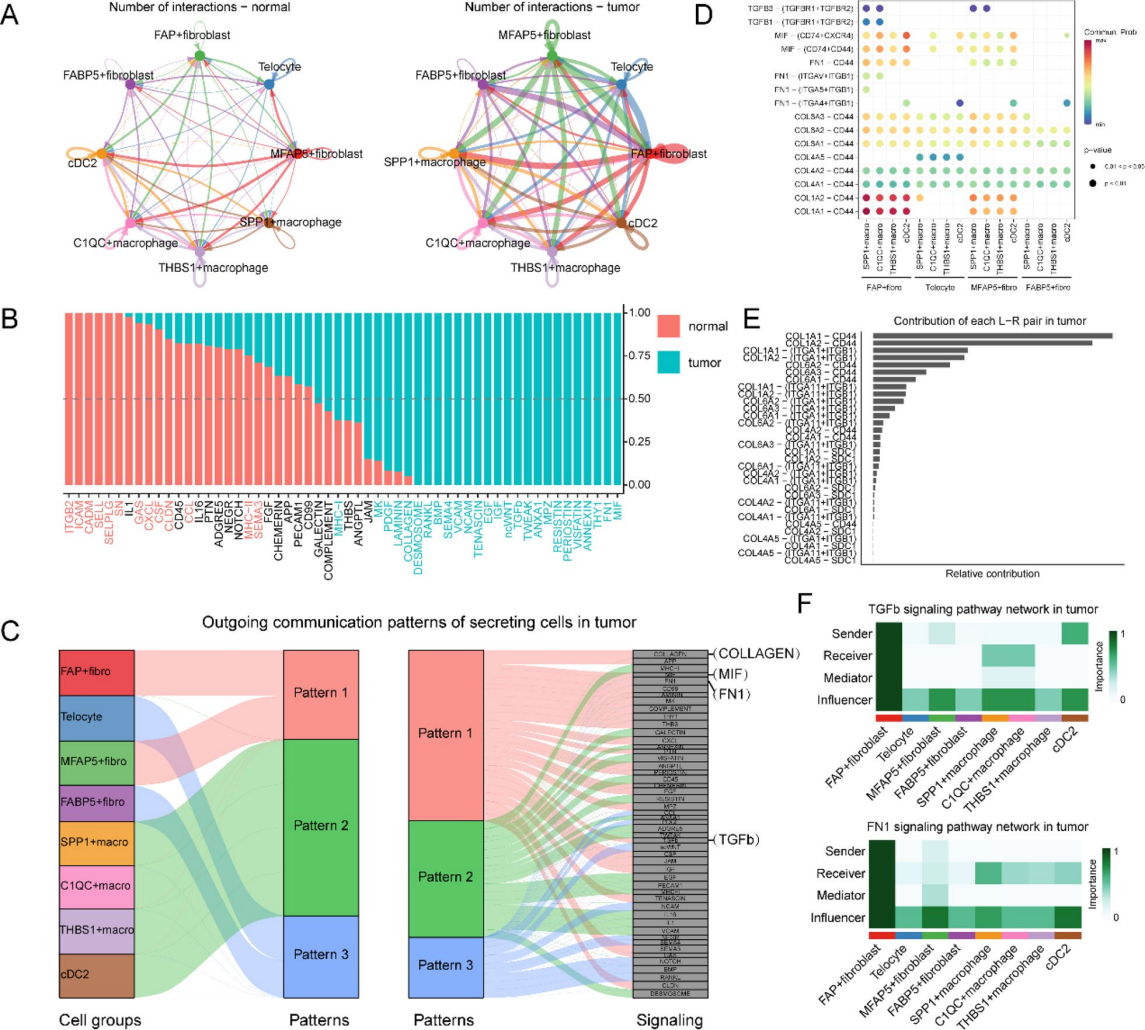
Fibroblasts-myeloid cells based regulatory network detect tumor-specific signaling pathways in CRC. **A** The circle plots show the overview of cell–cell interaction numbers between fibroblasts and myeloid cells in tumor tissues (right) and normal tissues (left) respectively, the broader arrow, the more numbers of interactions. **B** Bar plot of tumor- or normal- specific signaling pathways between fibroblasts and myeloid cells inferred by CellChat, red color represents the signals that increased in normal tissues, and blue color represents the interactions increased in tumor tissues. **C** The river plot shows the outgoing communication patterns of certain cells in CRC tissue. **D** Dot plot of ligand-receptor (L-R) pairs of several tumor-specific pathways between fibroblasts (sources) and myeloid cells (receptors). **E** Bar plot shows the contribution of all L-R pairs in COLLAGEN pathway within the tumor tissue. **F** Heatmap shows dominant senders, receivers, mediators and influencers in TGFβ and FN1 signals of tumor inferred by network centrality score

Multiple studies have demonstrated that far-ranging reciprocity exists between fibroblasts and myeloid cells. This reciprocity has far-reaching effects on the progression of cancers [5]. In order to clarify the cell–cell interaction mechanisms between these cells, we built separate intercellular communication networks between fibroblasts and myeloid cells in tumors (including the core and border) and normal tissues (Additional file 1: Tables S10–S13). As demonstrated in Fig. 5A, we observed that MFAP5 + and FAP + fibroblasts had the highest number of communications with other stromal and immune cells in the tumor, indicating their active biological properties. We observed that several pro-tumor signal pathways were primarily present between these cells, including MIF, FN1, TGF-β and COLLAGEN pathways (Fig. 5B). Outgoing communication pattern analysis also suggested a coordinated pro-tumor signaling pathway between FAP + and MFAP5 + fibroblasts, which have long been verified as a tumor-driven stromal population [41]. We extrapolated that MFAP5 + fibroblasts could drive tumor development by signal coordination with FAP + fibroblasts (Fig. 5C).

MIF signals are reportedly associated with the immunological escape from the TME. Our study highlights that tumor-associated fibroblasts (especially FAP + and MFAP5 + fibroblasts) can secrete MIF, which binds to the corresponding receptor CD74 on macrophages to drive immunological escape. This results in an immunosuppressive microenvironment in CRC (Fig. 5D) [42, 43]. Inhibition of the fibroblast-mediated MIF-CD74 signaling axis or the blocking of pro-tumor fibroblasts has the potential to eliminate tumor immunosuppression. Regarding the COLLAGEN pathway, we observed that MFAP5 + fibroblasts highly expressed collagen (encoded by COL1A1, COL1A2 and COL6A2 et al.) to interact with the integrin receptors, CD44 and SDC1 of myeloid cells (Fig. 5D, E). This phenomenon indicates that fibroblast-released collagen may interact with the relevant receptors of myeloid cells to prompt the formation of the extracellular matrix (ECM). This results in a desmoplastic niche, thus modulating tumor growth. In addition, tumor-specific MFAP5 + fibroblasts could also secrete FN1 and TGF-β to interact with certain receptors of myeloid cells to promote tumor progression (Fig. 5D and F) [24]. Overall, our findings emphasize that MFAP5 + fibroblasts and myeloid cells exhibit complicated intercellular interactions that aggravate tumor malignancy. The targeting of tumor-specific cell–cell networks could be a promising strategy for CRC treatment.

### MFAP5 + fibroblasts modulate the phenotype of C1QC + macrophages through the IL34/CSF1R signal pathway

**Fig. 6:**
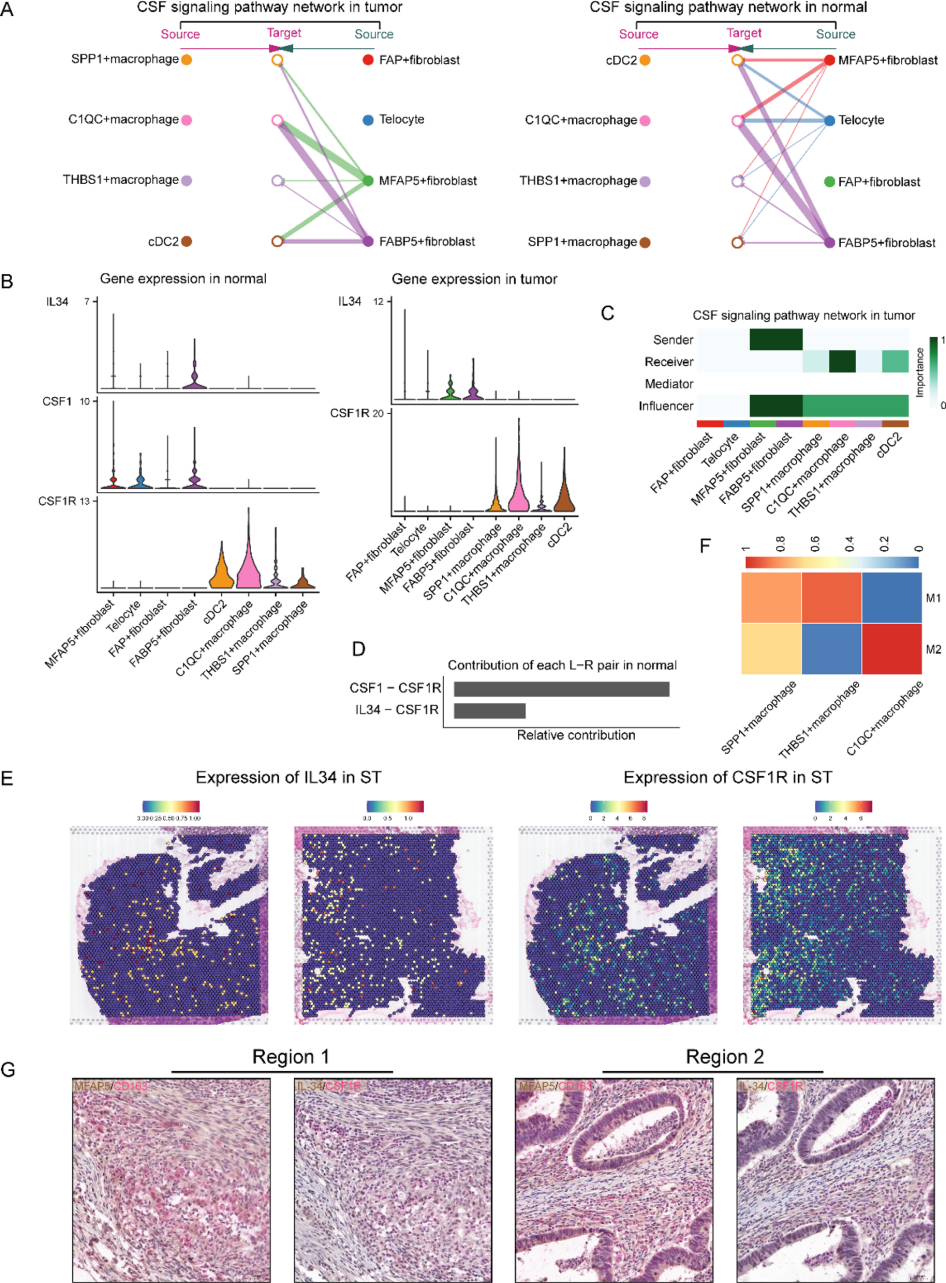
MFAP5 + fibroblasts modulate the phenotype of C1QC + macrophages through the IL34/CSF1R signal pathway. **A** The hierarchy plots of CSF signaling pathway network indicate the regulation of MFAP5 + fibroblasts and FABP5 + fibroblasts on myeloid cells in normal or tumor sites. **B** Violin plot of IL34, CSF1, and CSF1R expression of tumor-specific fibroblasts and myeloid cells in distinct tissues (normal or tumor). **C** Heatmap shows dominant senders, receivers, mediators and influencers in CSF signals of tumor inferred by network centrality score. **D** Bar plot shows the contribution of all L-R pairs in CSF pathway within the normal tissue. **E** Spatial images localize the expression of IL34 and CSF1R which suggest that the co-localization of IL34 and CSF1R in CRC slices. **F** Heat map of M1/M2 phenotype scores of three identified tumor-associated macrophage subtypes. **G** Immunohistochemical double-staining of MFAP5/CD163 and IL-34/CSF1R using continuous slides of CRC tissues (×20, 50 μm)

Macrophages are important innate immune cells that play a crucial role in maintaining homeostasis [44]. Macrophage proliferation and differentiation depend on the release of specific cytokines and growth factors, including Colony Stimulating Factor-1 (CSF1) and interleukins [44]. Our intercellular interaction analyses demonstrated that the CSF pathway is vital for cellular signaling in tumor-associated fibroblast-macrophage-mediated regulatory networks in tumor tissues. We also observed that FABP5 + fibroblasts perform primary functions on C1QC + macrophages in normal tissues through CSF1-CSF1R/IL34-CSF1R. However, in tumor tissues, C1QC + macrophages receive CSF signaling from both MFAP5 + and FABP5 + fibroblasts, suggesting an immunomodulatory role in CRC (Fig. 6A–D). Moreover, when we visualized the expression of ligands and receptors of the CSF/IL34/CSF1R signaling axis, we observed that MFAP5 + fibroblasts in normal tissues mainly secreted CSF1 to interact with C1QC + macrophages. However, in tumors, MFAP5 + fibroblasts could only release IL-34, which is another cytokine that binds with C1QC + macrophages through the receptor CSF1R (Fig. 6B). We subsequently applied ST data to validate the existence of the IL34-CSF1R signal axis between these cells in CRC (Fig. 6E). Accumulating evidence demonstrated that IL-34 favors the differentiation of M2-phenotype macrophages in diverse cancers by binding to CSF1R [44, 45]. Our analyses (Fig. 6F) and previously published studies both suggest that C1QC + macrophages demonstrate high score of M2 signature [46]. Double-staining IHC of MFAP5/CD163 and IL-34/CSF1R of continuous CRC slices suggested the existence of IL-34/CSF1R axis between MFAP5 + fibroblasts and M2-type macrophages (Fig. 6G). While this phenotype might be associated with tumor immune escape, we hypothesized that the change from CSF1 to IL-34 could be important during the MFAP5 + fibroblasts mediated promotion of tumor malignancy through the driving of the immune evasive phenotype of C1QC + macrophages. Moreover, IL-34 depletion could be an optimal strategy to block this process [45, 47]. In the future, in-depth basic research should be conducted to identify the key genes involved in this process and to develop new therapies to block this signaling pathway and inhibit intra-tumor suppressive immunity in CRC.

### Reciprocally pro-tumorigenic crosstalk between MFAP5 + fibroblasts and myeloid cells supports tumor progression

**Fig. 7:**
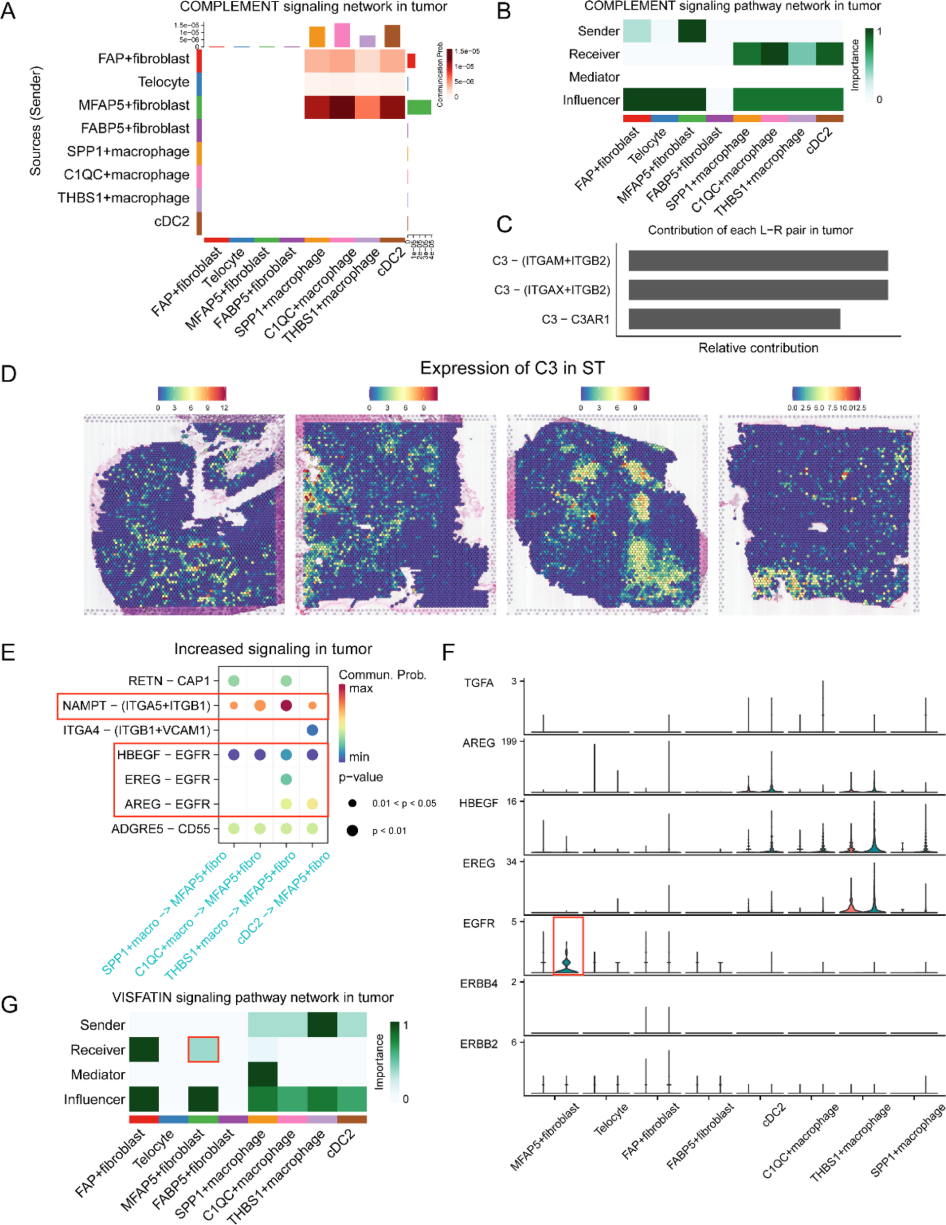
Reciprocally pro-tumorigenic crosstalk between MFAP5 + fibroblasts and myeloid cells support tumor progression. **A** Heatmap of COMPLEMENT signal suggest the key role of MFAP5 + fibroblasts in regulating the functions of C1QC + macrophages and other immune cells in tumor. **B** Heatmap shows dominant senders, receivers, mediators and influencers in COMPLEMENT signals of tumor inferred by network centrality score. **C** Bar plot shows the contribution of all L-R pairs in tumor COMPLEMENT pathway. **D** Spatial transcriptomics visualize the C3 expression at spatial tissue architecture. **E** Dot plot shows the increased signals that tumor-infiltrating myeloid cells could secreted to target MFAP5 + fibroblasts. **F** Violin plot shows the expression of L-R pairs of EGF signaling pathway between fibroblasts and myeloid cells in normal (red color) or tumor (green color). **G** Heatmap shows dominant senders, receivers, mediators and influencers in VISFATIN signals of tumor inferred by network centrality score

C1QC + macrophages are tumor-associated macrophages with complement activation, antigen processing and presentation [10]. However, the specific signaling network that regulates the function of C1QC + macrophages remains unclear as yet. We demonstrated that MFAP5 + fibroblasts and C1QC + macrophages are closely related. We subsequently investigated whether MFAP5 + fibroblasts could modulate the immunomodulatory functions of C1QC + macrophages. We observed that MFAP5 + fibroblasts were the main senders that could release complement C3 to interact with the receptors ITGAM/ITGB2, ITGAX/ITGB2, and C3AR1 of C1QC + macrophages in tumor tissues, thus activating the complement system (Fig. 7A–C). In order to confirm this phenomenon, we visualized the spatial expression of C3 in colorectal cancer. Our data suggested that C3 was co-localized with these cells (Fig. 7D). Hence, MFAP5 + fibroblasts might aberrantly activate the complement system of C1QC + macrophages to regulate tumorigenesis, development, and metastasis [48–50].

**Fig. 8:**
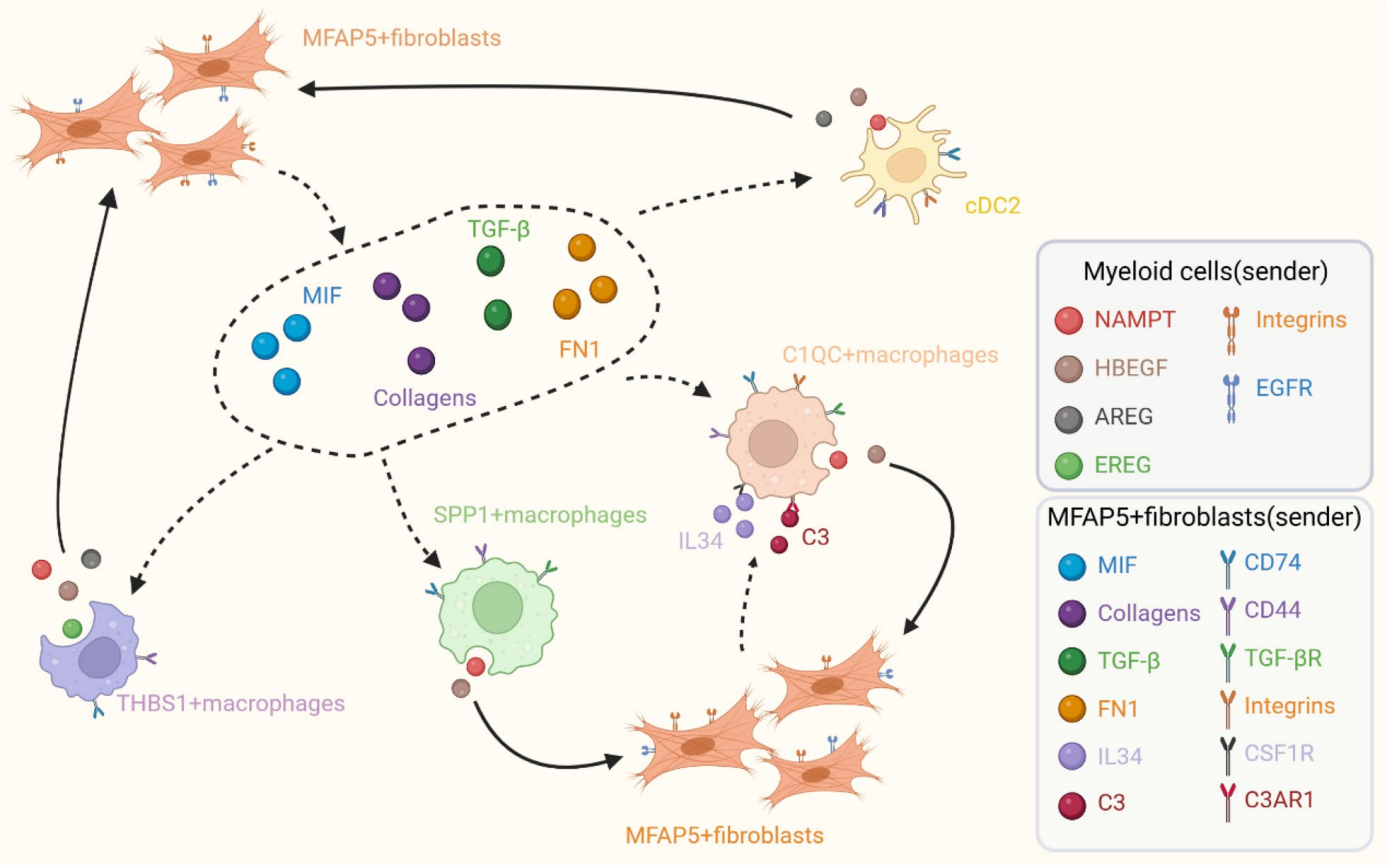
Cell-to-cell communication networks between MFAP5 + fibroblasts and tumor-infiltrating myeloid cells in CRC TME. In our analyses, we demonstrated that MFAP5 + fibroblasts have mutual interactions with C1QC + macrophages and other myeloid cells. In detail, MFAP5 + fibroblasts could release ligands include MIF, TGF-β, FN1 and Collagens to bind with corresponding receptors of myeloid cells so as to remodel the unfavorable conditions, besides, MFAP5 + fibroblasts could significantly interact with C1QC + macrophages through IL34/CSF1R and C3/C3AR1/(ITGAX + ITGB2)/(ITGAM + ITGB2) axes to module C1QC + macrophages phenotype and its immunomodulatory functions in tumor tissues. Reciprocally, myeloid cells could also secrete various factors to reshape the malignant behaviors of MFAP5 + fibroblasts by the EGF and VISFATIN signals

In addition to the regulatory functions of MFAP5 + fibroblasts, we examined the signal communication mediated by myeloid cells. As demonstrated in Fig. 7E, EGF signaling was uniquely upregulated in tumor-localized MFAP5 + fibroblasts. Myeloid cells secrete epidermal growth factor family proteins (HBEGF, AREG, and EREG) that can bind to the EGFR receptor. This receptor is specifically expressed in MFAP5 + fibroblasts. It modulates downstream signal transduction pathways (Fig. 7F). EGFR overexpression has been recognized as a key factor in tumor growth, angiogenesis, metastasis, and therapy resistance [51, 52]. Therefore, the inappropriate activation of EGFR in tumor-localized MFAP5 + fibroblasts could partly account for its malignant behavior. Furthermore, myeloid cells can transmit cancer-promoting signals to MFAP5 + fibroblasts via the NAMPT-ITGA5/ITGB1 pair (VISFATIN signaling pathway) (Fig. 7G) [53, 54]. In summary, reciprocal interactions between MFAP5 + fibroblasts and myeloid cells activate the immunosuppressive features of tumor-infiltrating myeloid cells (especially C1QC + macrophages), in addition to enhancing the aggressive phenotypes of MFAP5 + fibroblasts through a series of cancer-associated pathways. This makes up a positive loop that promotes tumor invasion in patients with colorectal cancer (Fig. 8).

## Discussion

**Fig. 9:**
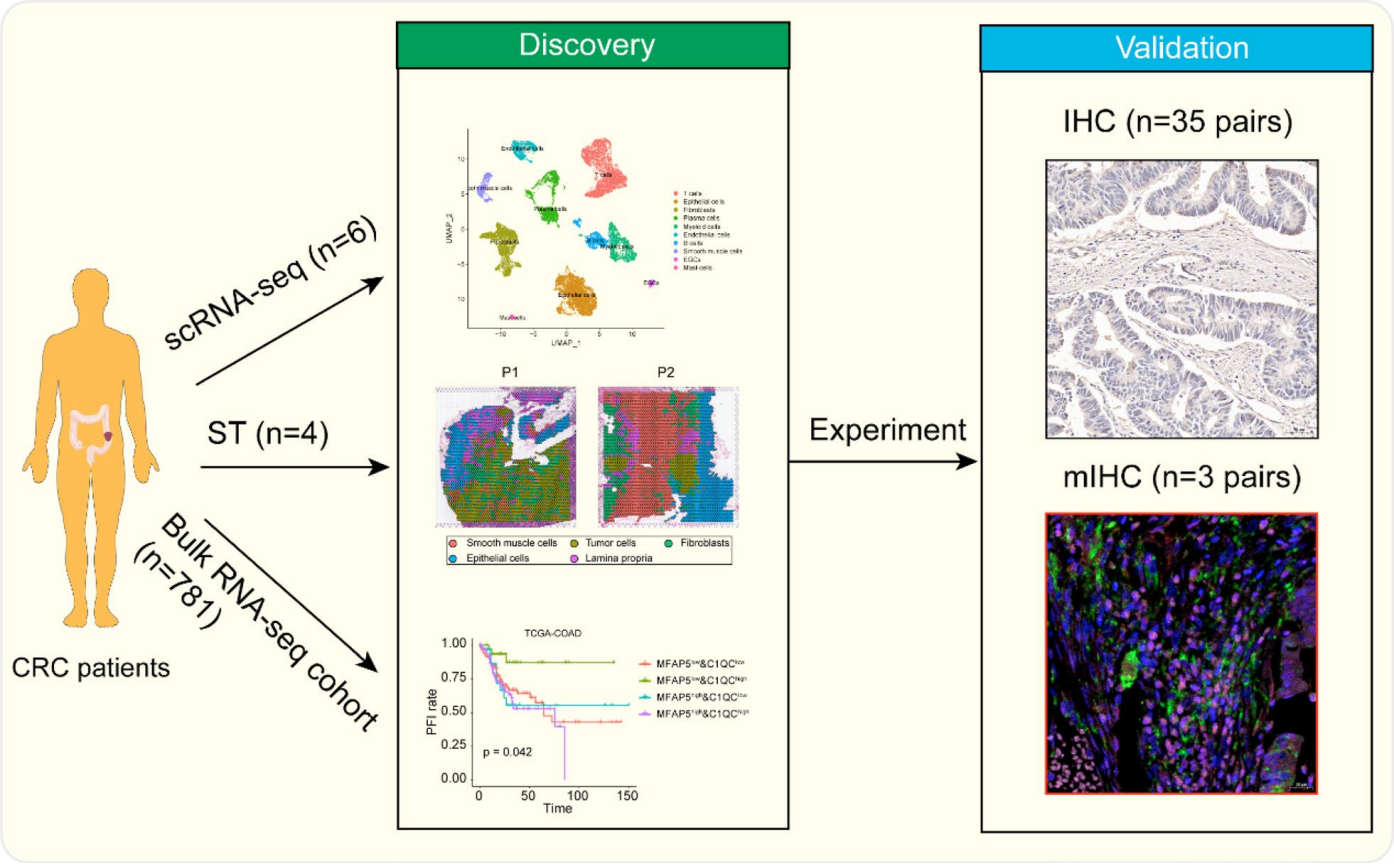
Workflow of this project. In this work, we combined bioinformatic analysis and experimental validation to explore the biological properties of MFAP5 + fibroblasts, with specially emphasize on its interactions with tumor-infiltrating C1QC + macrophages and other myeloid cells in CRC TME. scRNA-seq (n = 6 pairs, tumor core, border, and adjacent normal tissues), ST (n = 4, tumor tissues) and public cohort (COAD: n = 285, READ: n = 93, GSE14333: n = 226, GSE17536: n = 177) were integrated for comprehensive analyses of MFAP5 + fibroblasts at diverse perspective. Then, IHC (n = 35 pairs, tumor and normal tissues) was used to explore the existence of MFAP5 + fibroblasts as well as its correlation with clinical prognosis. mIHC (n = 3 pairs, tumor and normal tissues) was applied for investigating the crosstalk between MFAP5 + fibroblasts and C1QC + macrophages in patients with CRC

Recent studies have increasingly demonstrated the critical role of TME in CRC development, metastasis, and response to therapies [2]. Strategies to therapeutically target the TME are considered a promising approach for precise tumor treatment [55]. However, previous therapeutic strategies still fail to suppress tumor progression because of the complexity of TME components and far-ranging intercellular interactions. There is an urgent need to identify the interactions among tumor-specific cell populations that perform important functions in CRC tissues. In recent years, with the emergence of single-cell omics and spatial transcriptomics techniques, the details of TME biology have been explored in different cancer types. Many cell-to-cell communication networks have been associated with tumor malignancy [6]. Fibroblasts and myeloid cells are the most abundant stromal cells in solid tumors. These cells exhibit extensive crosstalk with each other [7]. An in-depth understanding of these interactions could provide a basis for therapeutic schemes and drug development. Therefore, we integrated scRNA-seq, ST, bulk RNA-seq, and basic experiments to decipher the cell–cell interactions of fibroblasts and myeloid cells, both at single-cell resolution and spatial organization (Fig. 9).

Fibroblasts are heterogeneous clusters that have been identified in different tumor types, stages, and metastatic organs [32]. Recently, many novel fibroblast subclusters have been identified with diverse gene signatures and functions. For example, we previously confirmed the existence of inflammatory-cancer-associated fibroblasts (iCAFs) and myo-cancer-associated fibroblasts (mCAFs) in CRC [38]. These have been considered as key factors for the remodeling of the TME into immunosuppressive niche [56, 57]. Other studies have identified other fibroblasts with a high expression of CD146 [58], α-SMA [59], FAP [24, 59] and Meflin [60], that exhibit tumor suppression or promotion tendencies. In this study, we focused on the key role of newly identified MFAP5 + fibroblasts in CRC tissues. Although there is evidence that MFAP5 expression in the stroma is associated with bladder and breast cancer malignant behavior [40, 61], there is still a lack of direct evidence on how MFAP5 + fibroblasts mediate the remodeling of the TME in CRC. We explored the biological characteristics of MFAP5 + fibroblasts and observed paradoxical findings: MFAP5 + fibroblasts mainly exist in normal tissues. However, patients with higher infiltration of MFAP5 + fibroblasts in tumor regions experienced worse prognosis than those with lower infiltration. This suggests the existence of tumor-specific factors, such as cell-to-cell interactions, that could significantly impact the properties of MFAP5 + fibroblasts, thus prompting its pro-tumor role.

Accumulating evidence indicates that fibroblasts can interact with multiple immune components via the secretion of various chemokines, cytokines, and other biomolecules. This results in the formation of an immunosuppressive niche that facilitates tumor immune escape [5, 24]. Ke et al. [62] reported that MFAP5 was associated with immune infiltration in uterine leiomyosarcoma. We investigated the relationship between MFAP5 + fibroblasts and multiple immune cells, including both adaptive and innate immune cells. In order to ensure the authenticity of our results, we performed analyses on three independent databases and used different computational algorithms to explore this relationship, both in spatial tissue architecture and bulk public cohorts. Our findings demonstrated a remarkable correlation between MFAP5 + fibroblasts and macrophages for the first time. Our results suggest that fibroblasts can recruit monocytes and transform them into M2 phenotype macrophages or induce immune inhibition via multiple key molecules [5]. This might explain the unfavorable physiologies of MFAP5 + fibroblasts. However, considering that macrophages are heterogeneous tumor-infiltrating immune cells and their functions are largely correlated with the context of the surrounding microenvironment [63], more studies are required to investigate the tumor-specific macrophage subclusters that MFAP5 + fibroblasts mainly interact with. This could provide a more direct target for pro-tumor subsets. Using bioinformatic analysis and experimental validation, we confirmed the close proximity of MFAP5 + fibroblasts to C1QC + macrophages in CRC tissues. Previous studies have demonstrated that C1QC + macrophages are enriched with inflammatory signatures [10] which might be associated with good clinical outcome [41]. Survival analysis demonstrated that patients with high infiltration of MFAP5 + fibroblasts and C1QC + macrophages experience shorter survival times than those with low expression of MFAP5 + fibroblasts and high expression of C1QC + macrophages. Fundamental experiments also suggest that MFAP5 + fibroblast-derived microfibrils could attach to C1QC + macrophages in border stromal regions and prompt them to interact with MFAP5 + fibroblasts. These findings also prove that the interactions between MFAP5 + fibroblasts and C1QC + macrophages are important factors affecting malignant tumor phenotypes and resulting in worse outcomes.

Considering the crucial role of MFAP5 + fibroblasts in tumor-driven signaling pathways in the TME, we performed intercellular interaction analysis to understand the potential pro-tumorigenic mechanisms. Our results revealed several tumor-specific signals between MFAP5 + fibroblasts and myeloid cells, particularly C1QC + macrophages. This suggests that MFAP5 + fibroblasts could prompt the M2 phenotype of macrophages with the IL34/CSF1R axis [44], supporting the immunosuppressive niche through MIF/CD74 signaling [43]. This also results in the formation of a desmoplastic microenvironment via collagen pathways and other intercellular signaling that facilitates unfavorable conditions for anti-tumor immunity. In addition, the signal co-ordination between MFAP5 + fibroblasts and pro-tumorigenic FAP + fibroblasts [24, 32, 59] further supports the invasive CRC microenvironment. Reciprocally, activated myeloid cells regulate the activation of MFAP5 + fibroblasts in a positive feedback mode by secreting cancer-promoting ligands that contain EGF superfamily proteins and NAMPT. These communication patterns could be considered as new targets for future CRC treatment.

Our study has some limitations, including a small sample size. In this study, we investigated the biological properties of MFAP5 + fibroblasts in cancer tissues. However, our analyses were based only on a single tumor type. In addition, it is still not known whether MFAP5 + fibroblasts are conserved subclusters residing in distinct cancer types. Other studies have suggested that MFAP5 in the stroma is associated with cancer invasion in breast and bladder cancers. However, there is little direct evidence for the presence of such fibroblasts at single-cell resolution. Hence, further studies are required to elucidate the conserved features of MFAP5 + fibroblasts in various cancers.

## Conclusions

In this study, we combined scRNA-seq, ST, bulk RNA-seq, and basic experiments to explore the detailed landscape of the CRC microenvironment from different perspectives. Our results provide comprehensive information regarding the physiological functions of MFAP5 + fibroblasts, with emphasis on their cell-to-cell crosstalk with C1QC + macrophages and other tumor-infiltrating myeloid cells. This could provide targetable strategies to overcome unfavorable conditions for patients with CRC.

## Supplementary Information

**Additional file 1.** Figures S1–S11, Tables S1–13.

## Data Availability

The single-cell CRC dataset of colorectal cancer analyzed in the present study can be reviewed at the Gene Expression Omnibus (GEO: GSE144735) repository: https://www.ncbi.nlm.nih.gov/geo/query/acc.cgi?acc=GSE144735. The spatial transcriptomics dataset was downloaded from the website built by Wu et al. [12] at http://www.cancerdiversity.asia/scCRLM/. Bulk transcriptome dataset of colorectal cancer TCGA COAD and READ are available at https://xenabrowser.net/datapages/, GSE14333 at: https://www.ncbi.nlm.nih.gov/geo/query/acc.cgi?acc=GSE14333 and GSE17536 at: https://www.ncbi.nlm.nih.gov/geo/query/acc.cgi?acc=GSE17536.

## Abbreviations

CRC: Colorectal cancer
TME: Tumor microenvironment
ECM: Extracellular matrix
CAFs: Cancer-associated fibroblasts
scRNA-seq: Single-cell RNA sequencing
ST: Spatial transcriptomics
TCGA: The Cancer Genome Atlas
GEO: Gene Expression Omnibus
PCA: Principal component analysis
UMAP: Uniform Manifold Approximation and Projection
DEGs: Differentially expressed genes
GO: Gene Ontology
HE: Hematoxylin–eosin staining
ssGSEA: Single sample gene set enrichment analysis
NMF: Nonnegative matrix factorization
EGCs: Enteric glial cells
TMA: Tissue microarray
DCs: Dendritic cells
CSF1: Colony stimulating factor-1
iCAFs: Inflammatory-cancer-associated fibroblasts
mCAFs: Myo-cancer-associated fibroblasts
IHC: Immunohistochemistry
mIHC: Multiplex immunohistochemistry
DAB: Diaminobenzidine
